# Differences in pain treatment between surgeons and anaesthesiologists in a physician staffed prehospital emergency medical service: a retrospective cohort analysis

**DOI:** 10.1186/s12871-019-0683-0

**Published:** 2019-01-31

**Authors:** Stefan J. Schaller, Felix P. Kappler, Claudia Hofberger, Jens Sattler, Richard Wagner, Gerhard Schneider, Manfred Blobner, Karl-Georg Kanz

**Affiliations:** 1 Klinik für Anästhesiologie und Intensivmedizin, Klinikum rechts der Isar, School of Medicine, Technical University of Munich, Munich, Germany; 2 Klinik für Unfallchirurgie, Klinikum rechts der Isar, School of Medicine, Technical University of Munich, Munich, Bavaria Germany

**Keywords:** Prehospital pain management, Prehospital emergency medicine, Emergency physicians

## Abstract

**Background:**

Although pain treatment is an important objective in prehospital emergency medicine the incidence of oligoanalgesia is still high in prehospital patients. Given that prehospital emergency medicine in Germany is open for physicians of any speciality, the prehospital pain treatment may differ depending on the primary medical education. Aim of this study was to explore the difference in pain treatment between surgeons and anaesthesiologists in a physician staffed emergency medical service.

**Methods:**

Retrospective single centre cohort analysis in a physician staffed ground based emergency medical service from January 2014 until December 2016. A total of 8882 consecutive emergency missions were screened. Primary outcome measure was the difference in application frequency of prehospital analgesics by anaesthesiologist or surgeon. Univariate and multivariate logistic regression analysis was used for statistical analysis including subgroup analysis for trauma and acute coronary syndrome.

**Results:**

A total of 8238 patients were included in the analysis. There was a significant difference in the application frequency of analgesics between surgeons and anaesthesiologists especially for opioids (*p* < 0.001, OR 0.68 [0.56–0.82]). Fentanyl was the most common administered analgesic in the trauma subgroup, but significantly less common used by surgeons (*p* = 0.005, OR 0.63 [0.46–0.87]). In acute coronary syndrome cases there was no significant difference in morphine administration between anaesthesiologists and surgeons (*p* = 0.49, OR 0.88 [0.61–1.27]).

**Conclusions:**

Increased training for prehospital pain treatment should be implemented, since opioids were administered notably less frequent by surgeons than by anaesthesiologists.

**Electronic supplementary material:**

The online version of this article (10.1186/s12871-019-0683-0) contains supplementary material, which is available to authorized users.

## Background

Stabilization of vital functions and treatment of pain is essential in prehospital emergency medicine. Importantly, pain is the main indication for alerting the prehospital emergency medical service (EMS) in Germany [1]. Moreover, sufficient pain relief is a key marker of quality in health care supply. Nevertheless, insufficiently or even not treated pain referred to as oligoanalgesia, is a well-recognized problem in the prehospital setting [2, 3], in particular in trauma patients [4–7]. A meta-analysis has established major reasons for oligoanalgesia: (1) insufficient or impossible communication with the patient, (2) the physicians’ presumption that analgesia could cover clinical symptoms and hence mislead the clinical diagnosis in the emergency department, and (3) fear of side effects, especially respiratory depression by opioids [8]. Furthermore, oligoanalgesia is accompanied by low quality of pain documentation being unclear if the latter is reason or part of the avoidance strategy [7]. Finally, prehospital oligoanalgesia occurs in different countries and systems, regardless the organisation of the EMS system [2–5, 7].

In Germany, the EMS is staffed with paramedics and supported by physicians working in any patient caring discipline, who have completed a postgraduate training and examination in prehospital medicine. If the rescue centre decides, based on severity, a physician is needed, or the paramedic on scene calls for reinforcement, the physician is dispatched. Administration of analgesics is allowed to physicians only by federal law and forbidden for paramedics and nurses.

To work in prehospital care, physicians require a minimum clinical experience of 24 months in any patient caring department, including at least six months in anaesthesiology, intensive care medicine or an emergency department. In addition, treatment of 50 prehospital emergency patients under supervision of a responsible prehospital physician, and a preparation course of 80 h in prehospital emergency medicine is necessary [9]. Physicians, who have then passed the prehospital emergency care examination, continue to work in their primal specialty working some shifts in prehospital care only. Given that prehospital emergency medicine is open for physicians of any medical speciality with its specific training and its specific in-hospital standard for pain treatment, the prehospital pain treatment including the problem of oligoanalgesia may differ depending on the primary medical education.

Aim of this study, therefore, is the exploration of differences in pain treatment between surgeons and anaesthesiologists in a physician staffed EMS service with a focus on the two most frequent subgroups with severe pain and opioid administration: chest pain and trauma [10, 11].

## Methods

### Study setting

After Ethics Committee approval (Ethics Committee of the Medical Faculty of the Technical University of Munich, Munich, Germany, No. 59/15), data were obtained from all prehospital emergency physician standardized forms of one dispatch location (fire department 10) in Munich, Germany between January 1st, 2014, to December 31st, 2016. The ethical committee waived the requirement for informed consent. This ground based EMS is a special equipped BMW X3 (Bayerische Motorenwerke, Munich, Germany) staffed with a paramedic from the Munich fire department who also serves as driver and a physician working at a university hospital (Klinikum rechts der Isar, Technische Universität München, Munich, Germany) mainly of the specialities anaesthesiology or surgery. The BMW X3 is equipped with different analgesics according to Bavarian standards: acetaminophen, butylscopolamine, metamizole, acetylsalicylic acid, ketamine and the opioids fentanyl and morphine.

### Data points and definitions

Data was collected from standardized EMS forms (DIVI version 4.2 (see Additional file 1), DIVI version 5.0 (see Additional file 2) and DIVI version 5.1 (see Additional file 3) containing patient and field data. We stored and analysed anonymized data only: Gender, age, heart rate, intubation, Glasgow Coma Scale (GCS), pain assessment as numeric rating scale (NRS), National Advisory Committee for Aeronautics’ (NACA) severity score, disease categories based on protocol classification, suspected diagnosis, specialty of the performing physician, assessment of injury and administered drugs.

Trauma cases were defined as cases where injuries were assessed in the protocols. Acute coronary syndrome (ACS) cases were defined with documentation of a suspected diagnosis of instable angina pectoris, ACS, non-ST segment elevation myocardial infarction (NSTEMI) and ST elevation myocardial infarction (STEMI).

Primary outcome measures were selection of prehospital analgesics and administration frequency by anaesthesiologist or surgeon. Prehospital analgesics selection and frequency was chosen as a measurement for the difference in prehospital pain treatment between members of these two medical specialities.

We further assessed documentation quality, that means documentation frequency of GCS, NRS, patient sex, age, heart rate and NACA severity score.

### Statistical analysis

Statistical analysis was done in the anonymized dataset. Results are expressed as median [Interquartile Range (IQR)] or frequencies with counts and percentages as appropriate. For statistical calculation we used logistic regression for binary outcomes and t-test or Mann-Whitney test as appropriate. For data points with adequate documentation frequency (> 90%) univariate analysis was performed. If significant, the variable was included in the multivariate analysis. Analyses were performed with IBM SPSS Premium Statistics for Windows v24.0 (IBM Corporation 2016, USA). All statistical tests were based on a 0.05 significance level and presented with 95% confidence intervals (CI). A subanalysis was performed for trauma and ACS cases.

## Results

### Study population

A total of 8882 protocols during January 1st 2014 and December 31st 2016 were evaluated and consequently 644 excluded due to false alerts, treatment by one specialist of internal medicine or missing documentation of the treating physician (Fig. 1). Consequently, 8238 cases were included in the analysis, performed by anaesthesiologists and surgeons. Patient characteristics are presented in Table 1, physicians characteristics in Table 2.

**Table 1:** Patients Characteristics

Patient Characteristic	Study population (*n* = 8238)	Anaesthesiologists (*n* = 6492)	Surgeons (*n* = 1746)	*p*-values
Female, n (%)	3979 (48.7)	3131 (48.6)	848 (48.9)	0.84
Age, median (IQR)	64 (40–79)	64 (41–79)	63 (39–78)	0.12
Intubated, n (%)	280 (3.4)	212 (3.3)	68 (3.9)	0.20
GCS initial, median (IQR)	15 (14–15)	15 (14–15)	15 (14–15)	0.63
Disease Categories, n (%)				
Cardiovascular	2695 (32.7)	2106 (32.4)	589 (33.7)	0.31
subgroup ACS cases	947 (11.5)	761 (11.7)	186 (10.7)	0.21
Traumatic	1541 (18.7)	1199 (18.5)	342 (19.6)	0.29
subgroup Polytrauma	72 (0.9)	48 (0.7)	24 (1.4)	0.013
Neurologic	901 (10.9)	733 (11.3)	168 (9.6)	0.048
Respiratory	670 (8.1)	535 (8.2)	135 (7.7)	0.49
Visceral	564 (6.8)	449 (6.9)	115 (6.6)	0.63
Mental	495 (6.0)	385 (5.9)	110 (6.3)	0.56
Endocrinological	277 (3.4)	225 (3.5)	52 (3.0)	0.32
Paediatrical	185 (2.2)	144 (2.2)	41 (2.3)	0.75
Gynaecological/Obstetrical	54 (0.7)	44 (0.7)	10 (0.6)	0.63
Other	856 (10.4)	672 (10.4)	184 (10.5)	0.82

n (%), number (percentages), *IQR* Inter Quartile Range, *GCS* Glasgow Coma Scale, *ACS* acute coronary syndrome

**Table 2:** Physician characteristics

Physician characteristic		Study population (*n* = 8238)	Anaesthesiologists (*n* = 6492)	Surgeons (*n* = 1746)	p-value
Sex	female	2209 (26.8)	2209 (34.0)	0 (0.0)	n/a
	male	6029 (73.2)	4283 (66.0)	1746 (100)	
Qualification	resident	2574 (31.2)	2378 (36.6)	196 (11.2)	< 0.001
	specialist	5664 (68.8)	4114 (63.4)	1550 (88.8)	

n (%), number (percentages), n/a, not applicable

### Documentation quality

Most consistently documented was age (99%), sex (99%) and initial GCS (95%, Table 3). NACA score and NRS documentation was inadequate. Anaesthesiologists documented the NRS less often at the beginning (37.3% vs. 41.2%) but not in the end (25.8% vs. 27.4%) of their care of patients compared to surgeons. In the subgroups trauma and ACS, a higher compliance of NRS documentation for both disciplines was achieved, but was still poor in total. The highest documentation rate for NRS with above 50% documentation frequency was achieved if an opioid was administered.

**Table 3:** Documentation quality

	Study population (*n* = 8238)	Anaesthesiologists (*n* = 6492)	Surgeons (*n* = 1746)	p-value
GCS initial documented	7806 (94.8)	6143 (94.6)	1663 (95.2)	0.30
GCS end documented	3817 (46.3)	3133 (48.3)	684 (39.2)	< 0.001
NACA documented	3709 (45.0)	2787 (42.9)	922 (52.8)	< 0.001
Heartrate initial documented	6875 (83.5)	5392 (83.1)	1483 (84.9)	0.06
Heartrate end documented	4990 (60.6)	3932 (60.6)	1058 (60.6)	0.98
Age documented	8194 (99.5)	6456 (99.4)	1738 (99.5)	0.62
Sex documented	8177 (99.3)	6442 (99.2)	1735 (99.4)	0.55
NRS initial documented	3139 (38.1)	2419 (37.3)	720 (41.2)	0.002
trauma cases (*n* = 1541)	741 (48.1)	576 (48.0)	165 (48.2)	0.95
ACS cases (*n* = 947)	478 (50.5)	379 (49.8)	99 (53.2)	0.40
if pain drug was administered (*n* = 2067)	1112 (53.8)	904 (53.4)	208 (55.6)	0.44
if opioid was administered (*n* = 1287)	717 (55.7)	598 (54.7)	119 (61.7)	0.07
NRS end documented	2154 (26.1)	1676 (25.8)	478 (27.4)	0.19
trauma cases (n = 1541)	494 (32.1)	393 (32.8)	101 (29.5)	0.26
ACS cases (n = 947)	396 (41.8)	317 (41.7)	79 (42.5)	0.84
if pain drug was administered (n = 2067)	822 (39.8)	675 (39.9)	147 (39.3)	0.84
if opioid was administered (n = 1287)	554 (43.0)	468 (42.8)	86 (44.6)	0.65

n (%), number (percentages), *GCS* Glasgow Coma Scale, *NACA* National Advisory Committee for Aeronautics’ severity score, *NRS* numeric rating scale

### Pain medication use

There was no significant difference in the administration frequency between surgeons and anaesthesiologists for any non-opioid: ketamine (*p* = 0.27), butylscopolamine (*p* = 0.88), acetaminophen (*p* = 0.25) and metamizole (*p* = 0.34, see Additional file 4). This was different for opioids with a highly significant difference in the univariate analysis (*p* < 0.001, see Additional file 4). Multivariate analysis showed that surgeons significantly less often administered opioids compared to anaesthesiologists (*p* < 0.001, OR 0.68 [0.56–0.82]; Table 4) independent if the patient was intubated (p < 0.001), of the initial GCS (*p* < 0.001), the disease category (*p* < 0.001), the physician’s qualification (*p* = 0.004) and the physician’s sex (*p* < 0.001). In the subgroup of fentanyl administration, the difference remained significant (*p* < 0.001, OR 0.59 [0.46–0.77]; see Additional file 5), but not for morphine administration (*p* = 0.08; see Additional file 6).

**Table 4:** Multivariate analysis of opioid use

	OR_adj_ (95% CI)		
Factor	Total	Trauma	ACS
Surgeon	0.68 (0.56–0.82)	0.71 (0.52–0.96)	0.93 (0.64–1.34)
Physician qualification resident	1.23 (1.07–1.42)	–	1.80 (1.35–2.40)
Physician sex female	1.40 (1.20–1.63)	1.86 (1.42–2.44)	1.35 (1.00–1.83)
ACS	8.34 (7.01–9.91)	n/a	n/a
Trauma	5.93 (5.07–6.93)	n/a	n/a
Age > 65 yrs	–	1.77 (1.39–2.27)	–
Female Patient	–	1.20 (0.94–1.52)	–
Patient intubated	12.88 (8.67–19.11)	45.33 (12.92–159.05)	–
GCS < 13	0.29 (0.21–0.40)	0.07 (0.03–0.19)	–

Factors included in the multivariate analysis if *p* < 0.05 in univariate analysis, *n/a* not applicable, −, not significant in univariate analysis, *OR*_*adj*_ adjusted Odds-Ratio in multivariate analysis, *CI* Confidence Interval, yrs., years, *GCS* Glasgow Coma Scale, *ACS* acute coronary syndrome

Subsequently, two sub-cohorts were tested: trauma (Table 4, details in see Additional file 5) and ACS (Table 4, details in see Additional file 6). In the trauma subgroup, surgeons administered fentanyl significantly less often (*p* = 0.005, OR 0.63 [0.46–0.87], see Additional file 5). In the subgroup of ACS, morphine was administered in 40.2% (see Additional file 4). Although there was a trend that anaesthesiologists administered morphine more frequently (42.0%) than surgeons (32.8%) this was not significant in the multivariate analysis (*p* = 0.49, OR 0.88 [0.61–1.27], see Additional file 6).

## Discussion

Our study suggests that specialization influences pain treatment in prehospital emergency cases, in particular between surgeons and anaesthesiologists. Especially the selection of opioids, in particular fentanyl, was more likely administered by anaesthesiologists than by surgeons.

There is no general European or German guideline for pain treatment in the prehospital setting for trauma patients. The level 3 (S3) evidence- and consensus-based guideline on the treatment of patients with severe and multiple injuries published [12], includes recommendations for emergency anaesthesia only. US recommendations for treatment of prehospital trauma patients postulate that opioids should be used in moderate to severe pain, as long as there are no contraindications [13]. In our data, opioids were the most used analgesics, especially in trauma and ACS cases.

For ACS, however, distinct guidelines are available: According to the 2015 European Society of Cardiology (ESC) guidelines for NSTEMI patients, morphine administration is reasonable for patients with persisting severe chest pain if the ischaemic symptoms do not relieve by nitrates and beta-blockers [14]. This might explain why there was no difference in administration frequency of morphine between surgeons and anaesthesiologists. There were, however, concerns about administration of morphine and a probable delay on prasugrel and ticagrelor effect [15, 16]. Recently, it has been shown, that prehospital morphine use did not increase one-year mortality in STEMI patients [17]. In these studies, the frequency of morphine administration was much lower (32 and 19%, respectively) than in our study (40.2%), although prehospital morphine administration in STEMI patients is recommended [15, 17, 18].

Pain treatment and sedation is an inherent part of speciality trainings in anaesthesiology as well as in surgery [9]. While anaesthesiologists, however, use powerful analgesics like fentanyl and morphine permanently, e.g. for narcosis induction, postoperative treatment and sedation in the intensive care unit, the daily routine of surgeons requires more often the constant training of other skills. Hence, anaesthesiologists might have a higher self-confidence in dosing and treatment of possible complications after administration of strong analgesics like fentanyl [19, 20]. Typical complications from side effects of opioids in the prehospital setting are nausea, vomiting (with the possibility of aspiration), decrease of the respiratory drive, the respiratory rate or tidal volume. The consequence of the described respiratory effects may lead to hypoventilation and upper airway obstruction in susceptible individuals.

While surgeons and anaesthesiologists built a team in operating theatre and use their different abilities synergistically, they are alone in the emergency field, that leads to different views depending on their speciality. Mechanical skills like splinting and positioning of fractures might be a common attempt of pain treatment by surgeons, maybe more common compared to anaesthesiologists. This could explain the difference in the use of fentanyl was highly significant (*p* < 0.001, see Additional file 5). Unfortunately, it was not possible to determine physical interventions, due to bad documentation compliance in this point.

Nevertheless, actions should be implemented to improve surgeons’ prehospital pain treatment: (1) Sattler et al. [21] suggested, adding a weaker opioid like piritramide to the available drugs on the EMS system might be an option to improve pain treatment by surgeons who are used to administer such drugs on a daily basis on their wards. An additional weaker opioid, however, would have important disadvantages: the onset time is slower and the risk of oligoanalgesia in patients with moderate to severe pain would continue. (2) Tactical Combat Casualty Care Guidelines from the US army [22] advise medical personnel to use fentanyl lozenges for moderate to severe pain without shock or respiratory distress [23]. In our system fentanyl lozenges are not available, but might be an option to raise fentanyl administration. (3) The most successful option in our point of view is to increase the training for prehospital physicians in knowledge and use of titrating pain medication.

In our comparison, the physicians’ speciality influenced the frequency of pain treatment with opioids significantly. A study about prehospital care differences between male and female trauma patients in Stockholm showed, that nearly one third of these patients received analgesics [24]. In this study the majority of cases were performed by emergency medical technicians and registered nurses. This may explain why our frequency of trauma cases with at least one analgesic administered in our physician staffed EMS, is higher (49.8%, see Additional file 5). A similar phenomenon was determined by the comparison between different EMS systems in four countries. The paramedic based systems in Coventry and Richmond and also the physician staffed EMS in Cantabria (general practitioners or family doctors) administered significantly less drugs in chest pain cases than the EMS in Bonn staffed by anaesthesiologists only. In all patients with cardiac chest pain and in the subgroup of patients with severe pain, treatment was more effective by anaesthesiologists than in other EMS systems [25].

Frequency of pain assessment in our trauma cases (48.1%, Table 3) is comparable with published data, showing rates of pain assessment and opioid administration averaging about 50% and that patient condition affect the ability of providers to effectively and appropriately manage pain [7]. However, in our data there was no significant difference in the frequency of pain assessment in trauma cases between anaesthesiologists and surgeons. The underassessment and undertreatment of pain really seems to be an omnipresent problem. A mandatory handover sheet might be an option or electronic documentation might improve documentation and quality control in the future [26]. Maybe the application of mandatory fields will be useful, because incomplete documentation was associated with increased mortality [27].

Our study has some methodical limitations. First, data is collected on a single emergency service location in Munich, which is staffed by physicians of one university hospital only. Second, since the study is retrospective it cannot detect the cause for the differences in pain treatment between anaesthesiologists and surgeons. Furthermore, we do not have outcome parameters of the hospital stay or any questionnaires filled out by the physicians to learn more about potential consequences of different treatments. Third, although comparable with other published data, documentation compliance was low. Therefore, we were not able to calculate pain scores and changes in pain scores or vital signs after administration of pain medication or report side effects of pain medication used.

## Conclusion

In summary, surgeons administered less opioids in the prehospital setting than anaesthesiologists, especially in trauma cases. However, no difference could be detected for morphine administration in ACS. Training for prehospital physicians in knowledge and use of titrating pain medication should be increased.

## Additional files

Additional file 1: Standardized Bavarian EMS form for physicians DIVI version 4.2. (PDF 97 kb)

Additional file 2: Standardized Bavarian EMS form for physicians DIVI version 5.0. (PDF 626 kb)

Additional file 3: Standardized Bavarian EMS form for physicians DIVI version 5.1. (PDF 940 kb)

Additional file 4: Table Univariate analysis of pain medication use. Presented as no. (%), ACS, acute coronary syndrome; Opioids (total, fentanyl & morphine) and non-opioid pain medication (ketamine, butylscopolamine, acetaminophen and metamizole); n/a, not applicable. (PDF 44 kb)

Additional file 5: Table Multivariate analysis of Fentanyl use. Factors included in the multivariate analysis if *p* < 0.05 in univariate analysis; n/a, not applicable; −, not significant in univariate analysis; OR_adj_, adjusted Odds-Ratio in multivariate analysis; CI, Confidence Interval; *yrs*., years; GCS, Glasgow Coma Scale; ACS, acute coronary syndrome. (PDF 39 kb)

Additional file 6: Table Multivariate analysis of Morphine use. Factors included in the multivariate analysis if *p* < 0.05 in univariate analysis; n/a, not applicable; −, not significant in univariate analysis, *OR*_adj_ adjusted Odds-Ratio in multivariate analysis, *CI* Confidence Interval, *yrs.* years, *GCS* Glasgow Coma Scale, *ACS* acute coronary syndrome. (PDF 38 kb)

## Abbreviations

ACS: Acute coronary syndrome
CI: Confidence interval
EMS: Emergency medical service
GCS: Glasgow Coma Scale
IQR: Interquartile Range
NACA: National Advisory Committee for Aeronautics’
NRS: Numeric rating scale
NSTEMI: non-ST segment elevation myocardial infarction
OR: Odds-Ratio
STEMI: ST elevation myocardial infarction
yrs.: Years
