# Supplementary material for: Differences in pain treatment between surgeons and anaesthesiologists in a physician staffed prehospital emergency medical service: a retrospective cohort analysis
Source: BMC Anesthesiol. 2019 Jan 31;19:18. doi: 10.1186/s12871-019-0683-0 (PMC6357417; doi:10.1186/s12871-019-0683-0)
Supplement: Supplementary file 2 — Standardized Bavarian EMS form for physicians DIVI version 5.0. (PDF 626 kb) [file 12871_2019_683_MOESM2_ESM.pdf]

Dieses Protokoll ist Urheberrechtlich geschützt. Unveränderte Vervielfältigungen sind uneingeschränkt zulässig, jegliche kommerzielle Verwertung sowie jede Änderung bedarf der Genehmigung durch die DIVI (Deutsche Interdisziplinäre Vereinigung für Intensiv- und Notfallmedizin - www.divi.de)

Anaphylaktische Reaktion: 0° lokal begrenzte Hautreaktion, I° disseminierte Hautreaktion (Flush, Urtikaria), Schleimhautreaktionen, Allgemeinreaktion (Unruhe, Kopfschmerz), II° Kreislaufdysregulation, Luftnot, Stuhl- und Urindrang, III° Schock, Bronchospasmus, Dyspnoe, Bewusstseinseintrübung, IV° Atem-, Kreislaufstillstand

GCS für Kinder: **Augen öffnen** [4] spontan [3] auf Schreien [2] auf Schmerzreiz [1] keine Reaktion - **Beste verbale Kommunikation** [5] Plappern, Brabbeln [4] Schreien tröstbar [3] Schreien, untröstbar [2] Stöhnen, unverständliche Laute [1] keine Reaktion - **Beste motorische Reaktion** [6] Spontane Bewegungen [5] auf Schmerzreiz gezielt [4] auf Schmerzreiz abnorme Abwehr [3] Beugeabwehr [2] Strecksynergismen [1] keine Reaktion

Pat-Stammdaten (ggf. mit Aufkleber überkleben / überdrucken)

Name

Vorname

Geb.Dat.

Straße

Plz, Ort

Kasse / Nr.

Vers. Nr.

Geschlecht ○ männlich ○ weiblich | BMI ○ ≤ 40 ○ > 40 (Gewicht [kg] / (Größe [m])²) | Alter Jahre ○ 1-7 Tg ○ 8-28 Tg

NOTARZT-EINSATZPROTOKOLL Version 5.0

Empfehlung der DIVI 2013 (www.divi.de)

○ Einsatz Nr. ○ Pat. Nr. | Pers Nr.

Standort

Einsatztechnische Daten

Einsatz-Datum

Einsatz-Ort

○ Wohnung ○ Sportstätte ○ Krankenhaus ○ Geburtshaus ○ Arbeitsplatz ○ Arztpraxis ○ Schule ○ Altenheim ○ Bildungseinrichtung ○ Massenveranstaltung ○ öff. Raum ○ Straße ○ Sonstige

Einsatz-Art

○ Primäreinsatz ○ Folgeeinsatz ○ Fehleinsatz ○ Notarzt-Nachforderung ○ Sekundäreinsatz z.B. Verlegung vorsorgliche Bereitstellung

Einsatzabbruch: ○ kein Patient ○ abbestellt ○ Pat. bereits abtransportiert ○ böswillige Alarmierung ○ technische Gründe ○ Wetter ○ Sonstige

Transportziel

☐ Voranmeldung ○ Stroke Unit ○ Herzkatheter ○ Traumazentrum

Beteiligt ☐ NEF ☐ NAW ☐ RTW ☐ KTW ☐ RTH ☐ ITH

Symptom-Beginn (z.B. Kollaps Unfallzeitpunkt) ☐ vor über 24h ☐ Kollaps beobachtet

Alarm

Ankunft (Einsatzort)

Ankunft (am Pat.)

Abfahrt

Übergabe ○ Krankenhaus ○ arztbesetztes Rettungsmittel ○ Sonstige

Einsatzbereit

Ende

Notarzt ○ Arzt in Weiterb. ○ Facharzt ○ Andere → ☐ Anästhesie ☐ Chirurgie ☐ Innere ☐ Pädiatrie ☐ Allg. Medizin ☐ Zusatz Intensiv

Assistenz ○ Rett.Ass. ○ Rett.San. ○ Notfall San. ○ (Int.)Pfleg.

RTW-Team

Rufname eigenes Fahrzeug

Notfallgeschehen, Anamnese, Erstbefund, Vormedikation, Vorbehandlung

AZ des Pat. vor dem Ereignis ○ gesund ○ leicht eingeschränkt ○ schwer eingeschränkt ○ lebensbedrohlich erkrankt ○ unbekannt

Ersthelfermaßnahmen (Laien) ○ suffizient ○ insuffizient ○ keine

First Responder ☐ vor Ort - ggf. Uhrzeit des Eintreffens

Erstbefunde - Neurologie

○ ohne path. Befund | Zeitpunkt

Augen öffnen
- spontan (4)
- auf Aufforderung (3)
- auf Schmerzreiz (2)
- kein Augenöffnen (1)

beste verbale Reaktion
- konversationsfähig orientiert (5)
- desorientiert (4)
- inadäquate Äußerungen (3)
- unverständliche Laute (2)
- keine (1)

beste motorische Reaktion
- folgt Aufforderung (6)
- gezielte Abwehr (5)
- ungezielte Abwehr (4)
- Beugesynergismen (3)
- Strecksynergismen (2)
- keine (1)

Arm / Bein – re / li

Zur Summierung der GCS wird nur der Wert der Extremität mit der besseren motorischen Reaktion herangezogen

Kinder-GCS-Definition siehe Blattrand

Glasgow Coma Scale (Summe)

Bewusstseinslage: ○ wach ○ getrübt ○ bewusstlos ☐ Reakt. auf Ansprache ☐ Reakt. auf Schmerzreiz ☐ analgosediert / Narkose

Stroke-Scoring: ○ nicht beurteilbar ☐ Seitenzeichen (Pupillen, periph. Motorik) ☐ kein Lächeln ☐ Sprachstörung ☐ Sehstörung ☐ Demenz ☐ Querschnittssymptomatik ☐ Babinski Zeichen ☐ Meningismus ☐ vorbestehende neurologische Defizite

Pupillenweite: rechts ○ eng ○ mittel ○ weit ○ entrundet; links ○ eng ○ mittel ○ weit ○ entrundet

Lichtreaktion: rechts ○ prompt ○ träge ○ keine; links ○ prompt ○ träge ○ keine

Messwerte initial

○ keine | Schmerzen 0 1 2 3 4 5 6 7 8 9 10

RR / | HF ○ rhythmisch ○ arrhythmisch | BZ ○ mg/dl ○ mmol/l

AF | SpO_2 ○ bei Raumluft ○ unter O_2-Gabe | Temp | $etCO_2$

EKG ○ kein EKG: ○ Sinusrhythmus ○ Abs. Arrhythmie ○ AV-Block II° ○ AV-Block III° ○ Schrittmacherrhythmus ○ Kammerflimmern ○ PEA / EMD ○ Asystolie ☐ STEMI ☐ schmale QRS-Tachykardie ☐ breite QRS-Tachykardie ☐ SVES / VES

Atmung ○ nicht US: ○ unauffällig ○ Dyspnoe ○ Schnappatmung ○ Apnoe ○ Beatmung ☐ Stridor ☐ Zyanose ☐ Spastik ☐ Hyperventilation ☐ Atemwegsverlegung ☐ Rasselgeräusche ○ sonstiges path. Atemmuster (Biot, Cheyne Stokes etc.)

Haut ○ nicht US: ○ unauffällig ○ pathologisch ☐ stehende Hautfalten ☐ Dekubitus ☐ Exanthem ☐ Oedeme ☐ kaltschweißig

Psyche ○ nicht US: ○ unauffällig ○ nicht beurteilbar ☐ wahnhaft ☐ erregt ☐ depressiv ☐ verwirrt ☐ aggressiv ☐ ängstlich ☐ suizidal ☐ verlangsamt/stuporös ☐ euphorisch ☐ motorisch unruhig

Erkrankungen

○ keine | ☐ Besiedelung mit multiresistenten Keimen vorbekannt (z.B. MRSA, MRSE, ESBL) ☐ abgedeckt offen

ZNS: ☐ Schlaganfall, TIA, intrakranielle Blutung ☐ im Lysefenster (Symptombeginn erfassen!) ☐ Krampfanfall ☐ Status epilepticus ☐ Meningitis / Encephalitis ☐ Synkope ☐

Herz-Kreislauf: ☐ Akutes Koronarsyndrom ☐ STEMI ☐ Vorderwand ☐ Hinterwand ☐ Rhythmusstörung ☐ tachy ☐ brady ☐ Lungenembolie ☐ orthostatische Fehlregulation ☐ Herzinsuffizienz ☐ Lungenödem ☐ Hypertensiver Notfall / hypertensive Krise ☐ Kardiogener Schock ☐ Schrittmacher- / ICD-Fehlfunktion ☐

Atmung: ☐ Asthma (Anfall) ☐ Status asthmaticus ☐ COPD (ggf. Exazerbation) ☐ Heimbeatmung ☐ Heimsauerstoff ☐ Pneumonie, Bronchitis ☐ Hyperventilationssyndrom ☐ Aspiration ☐ Haemoptysen ☐

Abdomen: ☐ Akutes Abdomen ☐ GI-Blutung ☐ obere ☐ untere ☐ Kolik (z.B. Niere, Galle) ☐ Enteritis ☐

Psychiatrie: ☐ Psychose, Manie, Erregungszustand ☐ Angst, Depression ☐ Intoxikation → ☐ akzidentell ☐ Alkohol ☐ Drogen ☐ Medikamente ☐ Sonstiges ☐ Entzug, Delir ☐ Suizid(versuch) ☐ Psychosoziale Krise ☐

Stoffwechsel: ☐ Hypoglykämie ☐ Hyperglykämie ☐ Exsiccose ☐ Urämie/ANV ☐ bek. Dialysepflichtig ☐

Pädiatrie: ☐ Fieberkrampf ☐ Pseudokrupp ☐ SIDS / Near-SIDS ☐

Gynäkologie: ☐ Schwangerschaft ☐ (drohende bzw. präklinische) Geburt ☐ (Prä-)Eklampsie ☐ vaginale Blutung ☐

Sonstige: ☐ anaphylakt. Reaktion ☐ I-II° ☐ III-IV° ☐ Hitzeerschöpfung, Hitzschlag ☐ Unterkühlung / Erfrierung ☐ hochfieb. Infekt / Sepsis / sept. Schock ☐ soziales Problem (ohne psych. Störung) ☐ medizinische Behandlungskomplikation ☐

Verletzungen

○ keine | Zusammenhang mit sportlicher Aktivität ○ Ja ○ Nein; beruflicher Aktivität ○ Ja ○ Nein

	leicht	mittel	schwer	geschlossen	offen
Schädel-Hirn	☐	☐	☐	☐	☐
Gesicht	☐	☐	☐	☐	☐
Hals	☐	☐	☐	☐	☐
Thorax	☐	☐	☐	☐	☐
Abdomen	☐	☐	☐	☐	☐
Wirbelsäule	☐	☐	☐	☐	☐
Becken	☐	☐	☐	☐	☐
Obere Extremitäten	☐	☐	☐	☐	☐
Untere Extremitäten	☐	☐	☐	☐	☐
Weichteile	☐	☐	☐	☐	☐

☐ Polytrauma.

☐ Verbrennung, Verbrühung ___ Grades ___ % ___ Grades ___ % ☐ Inhalationstrauma ☐ Elektrounfall ☐ (Beinahe-)Ertrinken ☐ Tauchunfall ☐ haemorrhagischer Schock

Unfallmechanismus: Trauma ○ stumpf ○ penetrierend; Sturz ○ <3m ○ >3m; Verkehr: Pat war ○ Fußgänger ○ Motorradfahrer ○ PKW-/LKW-Insasse ○ Fahrradfahrer ○ Sonstige; Gewaltanwendung ☐ Schlag ☐ Stich ☐ Schuss ☐ Sonstige

Erstdiagnosen

NACA SCORE INITIAL ○ I (geringfügige Störung) ○ II (ambulante Abklärung) ○ III (stat. Behandlung) ○ IV (Lebensgefahr nicht auszuschließen) ○ V (akute Lebensgefahr) ○ VI Reanimation ○ VII (Tod) ☐ Palliative Situation

Verlaufsbeschreibung

Zeit / Maßnahmen

Puls, RR, HDM, Defi, Transport T-T, Intubation, Injektion bei Rea: Suprarenin S, Amiodaron A

Skala: ≥220, 200, 180, 160, 140, 120, 100, 80, 60, ≤40

Werte über 220 bzw. unter 40 werden in der obersten bzw. untersten Zeile als Zahl zusammen mit dem zugehörigen Zeichen für Blutdruck oder Puls (∨ bzw. ○) als Zahl eingetragen

O2 [l/min]

SpO2 [%]

etCO2 [mmHg]

Medikation

○ keine Medikation

Infusionen	Handelsname	Dosis
Kristalloide Lsg.		ml
Kolloidale Lsg.		ml
hyperosmol. Lsg.		ml
Lipid		ml

Katecholamine	Handelsname	Dosis
Adrenalin		mg
Noradrenalin		mg

Sonstige	Handelsname	Dosis
Heparin		IE
Steroid		mg
Diuretikum		mg
Nitro		mg

Kardiaka	Handelsname	Dosis
Amiodaron		mg
Atropin		mg

Analgetika	Handelsname	Dosis
ASS		mg

Lysetherapie ○ vor Kreislaufstillstand ○ während Kreislaufstillstand ○ nach ROSC

...Vorab-Gabe durch Rettungsdienst (Notkompetenz): ☐ Adrenalin ☐ Amiodaron ☐ Glucose ☐ Analgetikum ☐ Benzodiazepin ☐ Nitrospray ☐ ß-Mimetikum (inhal.) ☐ Sonstige

Reanimation / Tod / Todesfeststellung (ergänzende Angaben)

☐ Reanimation ○ ROSC im Verlauf ○ niemals ROSC ○ keine Reanimation ○ erfolglose Reanimation → ○ nicht gewünscht / Patientenverfügung ○ aussichtslose Grunderkrankung ○ Sonstige

Beginn Rea ○ Ersthelfer ○ First Resp. ○ Rettungsd. ○ Notarzt

☐ KH-Aufnahme ○ bei ROSC ○ laufende Reanimation

☐ **Tod** ☐ Todesfeststellung | Zeitpunkt ☐ Zeitpunkt nicht bestimmbar

Übergabe

○ keine | Zeitpunkt | Glasgow Coma Scale

RR / | HF ○ rhythmisch ○ arrhythmisch | BZ ○ mg/dl ○ mmol/l

AF | SpO_2 | Temp | $etCO_2$

Schmerzen 0 1 2 3 4 5 6 7 8 9 10

EKG ○ kein EKG: ○ Sinusrhythmus ○ Abs. Arrhythmie ○ AV-Block II° ○ AV-Block III° ○ Schrittmacherrhythmus ○ Kammerflimmern ○ PEA / EMD ○ Asystolie ☐ STEMI ☐ schmale QRS-Tachykardie ☐ breite QRS-Tachykardie ☐ SVES / VES

Atmung ○ nicht US: ○ unauffällig ○ Dyspnoe ○ Schnappatmung ○ Apnoe ○ Beatmung ☐ Stridor ☐ Zyanose ☐ Spastik ☐ Hyperventilation ☐ Atemwegsverlegung ☐ Rasselgeräusche ○ sonstige pathologische Atemmuster (Biot, Cheyne Stokes etc.)

Bewusstsein ○ nicht US: ○ wach ○ getrübt ○ bewusstlos ☐ Reakt. auf Ansprache ☐ Reakt. auf Schmerzreiz ☐ analgosediert / Narkose

Einsatzverlauf - Besonderheiten

○ Übernahme aus arztbesetztem Rettungsmittel
○ Übergabe an arztbesetztes Rettungsmittel
○ Transport ins Krankenhaus ☐ mit Arzt
☐ mindestens eine Klinik nimmt nicht auf
☐ Zwangsunterbringung
○ nur Untersuchung und Behandlung
○ Patient lehnt Transport ab
○ Patient nicht transportfähig
○ Therapieverzicht / -beschränkung bewusst
☐ Erhöhter Hygieneaufwand
☐ Aufwendige techn. Rettung
☐ Schwerlasttransport erforderlich
☐ LNA am Einsatz
☐ MANV ☐ Behandlung mehrerer Patienten

Übergabe

○ keine ○ Praxis ○ ZNA / INA ○ Stroke Unit ○ Intensivstation ○ OP direkt ○ Sonstige ○ Hausarzt / KV-Arzt vor Ort ○ Fachambulanz ○ Schockraum ○ Herzkatheterlabor ○ Allgemeinstation

Übergabe an (Name)

Massnahmen

Zugänge (Art / Ort / Größe, Anzahl)
☐ peripherer Zugang
☐ intraossäre Punktion
☐ Transnasal-Applikator ☐ Sonstige
☐ Zugang erschwert ○ > 2 Versuche ○ Zugang unmöglich → Verfahrenswechsel

Atemweg
☐ Sauerstoffgabe l/min ○ als Präoxygenierung
☐ Freimachen der Atemwege / Absaugen
☐ Masken-/Beutel-Beatmung ☐ Maskenbeatmung nicht möglich
☐ supraglottische Atemwegshilfe ○ Larynxmaske ○ Larynxtubus ○ Sonstige
☐ endotracheale Intubation ○ oral ○ nasal | Größe Ch
☐ fiberoptische / elektronische Intubationshilfe
☐ Koniotomie / chirurgischer Atemweg
☐ sonstiger Atemwegszugang
☐ Intubation erschwert | Anzahl Versuche

Beatmung
○ manuell ○ maschinell ○ kontrolliert ○ assistiert ○ NIV | FiO2 | AF | AMV | PEEP | P_{max}

Defibrillation
☐ AED vor Ort | Erstanwendung durch ○ Laien ○ Rettungsdienst ○ Arzt
☐ Defi ○ monophasisch ○ biphasisch
1. Defibrillation (Zeitpunkt) | 1. ROSC (Zeitpunkt)
Anzahl Defi insgesamt | $Energie_{max}$ Joule

Reanimation
☐ Herzdruckmassage ○ Feedbacksystem ○ mechanisches Thoraxkompressionssystem
☐ Aktive Kühlung ○ Infusion ○ Kühlpackungen ○ technisch
☐ Vorab: Telefonanleitung zur Reanimation

Sonstige
☐ Spezielle Lagerung ☐ Zervikalstütze ☐ Schocklagerung ☐ Vakuummatratze ☐ Schaufeltrage ☐ Inkubator ☐ Wärmeerhalt ☐ Sonstige ☐ Oberkörper Hochlagerung ☐ stabile Seitenlage ☐ Spineboard ☐ sitzender Transport
☐ 12-Kanal EKG ☐ Spritzenpumpe(n) ☐ Funkübermittlung ☐ invasiver RR ☐ externer Schrittmacher ☐ Ultraschall
☐ Verband ☐ Reposition ☐ Beckenschlinge
☐ Thoraxdrainage re. ☐ Thoraxdrainage li. ☐ Blasenkatheter ☐ Magensonde
☐ Entbindung ☐ Krisenintervention
☐ Sonstiges

Bemerkungen (z.B. Verlauf, Hausarzt, Notkompetenz-Massnahmen)

NACA-Score bei Einsatzende ○ I (geringfügige Störung) ○ II (ambulante Abklärung) ○ III (stat. Behandlung) ○ IV (Lebensgefahr nicht auszuschließen) ○ V (akute Lebensgefahr) ○ VI Reanimation ○ VII (Tod)

Unterschrift Notarzt

V.5.0 - 1.73 - NoVar - 031113
