# Supplementary material for: Differences in pain treatment between surgeons and anaesthesiologists in a physician staffed prehospital emergency medical service: a retrospective cohort analysis
Source: BMC Anesthesiol. 2019 Jan 31;19:18. doi: 10.1186/s12871-019-0683-0 (PMC6357417; doi:10.1186/s12871-019-0683-0)
Supplement: Supplementary file 4 — Table Univariate analysis of pain medication use. Presented as no. (%), ACS, acute coronary syndrome; Opioids (total, fentanyl & morphine) and non-opioid pain medication (ketamine, butylscopolamine, acetaminophen and metamizole); n/a, not applicable. (PDF 44 kb) [file 12871_2019_683_MOESM4_ESM.pdf]

Table. Univariate analysis of pain medication use

	Study population	Anaesthesiologists	Surgeons	OR (95% CI)	P-Value
**Opioids**					
ACS	387 (40.9)	323 (42.4)	64 (34.4)	0.71 (0.51-0.99)	0.046
Trauma	484 (31.4)	406 (33.9)	78 (22.8)	0.58 (0.44-0.76)	<0.001
Total	1287 (15.6)	1094 (16.9)	193 (11.1)	0.61 (0.52-0.72)	<0.001
**Fentanyl**					
ACS	6 (0.6)	3 (0.4)	3 (1.6)	4.14 (0.83-20.69)	0.08
Trauma	458 (29.7)	388 (32.4)	70 (20.5)	0.54 (0.40-0.72)	<0.001
Total	689 (8.4)	590 (9.1)	99 (5.7)	0.60 (0.48-0.75)	<0.001
**Morphine**					
ACS	381 (40.2)	320 (42.0)	61 (32.8)	0.67 (0.48-0.94)	0.022
Trauma	27 (1.8)	19 (1.6)	8 (2.3)	1.49 (0.65-3.43)	0.35
Total	599 (7.3)	506 (7.8)	93 (5.3)	0.67 (0.53-0.84)	<0.001
**Ketamine**					
ACS	0 (0.0)	0 (0.0)	0 (0.0)	n/a	n/a
Trauma	203 (13.2)	163 (13.6)	40 (11.7)	0.84 (0.58-1.22)	0.36
Total	230 (2.8)	188 (2.9)	42 (2.4)	0.83 (0.59-1.16)	0.27
**Butylscopolamine**					
ACS	0 (0.0)	0 (0.0)	0 (0.0)	n/a	n/a
Trauma	0 (0.0)	0 (0.0)	0 (0.0)	n/a	n/a
Total	194 (2.4)	152 (2.3)	42 (2.4)	1.03 (0.73-1.45)	0.88
**Acetaminophen**					
ACS	0 (0.0)	0 (0.0)	0 (0.0)	n/a	n/a
Trauma	6 (0.4)	5 (0.4)	1 (0.3)	0.70 (0.08-6.01)	0.75
Total	92 (1.1)	68 (1.0)	24 (1.4)	1.32 (0.82-2.10)	0.25
**Metamizole**					
ACS	19 (2.0)	18 (2.4)	1 (0.5)	0.22 (0.03-1.68)	0.15
Trauma	222 (14.4)	169 (14.1)	53 (15.5)	1.12 (0.80-1.56)	0.52
Total	712 (8.6)	571 (8.8)	141 (8.1)	0.91 (0.75-1.10)	0.34
**At least one analgesic**					
ACS	403 (42.6)	338 (44.4)	65 (34.9)	0.67 (0.48-0.94)	0.020
Trauma	767 (49.8)	610 (50.9)	157 (45.9)	0.82 (0.64-1.04)	0.11
Total	2067 (25.1)	1693 (26.1)	374 (21.4)	0.77 (0.68-0.88)	<0.001

Presented as no. (%), ACS, acute coronary syndrome; Opioids, Fentanyl & Morphine; n/a, not applicable
