# Supplementary material for: Differences in pain treatment between surgeons and anaesthesiologists in a physician staffed prehospital emergency medical service: a retrospective cohort analysis
Source: BMC Anesthesiol. 2019 Jan 31;19:18. doi: 10.1186/s12871-019-0683-0 (PMC6357417; doi:10.1186/s12871-019-0683-0)
Supplement: Supplementary file 5 — Table Multivariate analysis of Fentanyl use. Factors included in the multivariate analysis if p < 0.05 in univariate analysis; n/a, not applicable; −, not significant in univariate analysis; ORadj, adjusted Odds-Ratio in multivariate analysis; CI, Confidence Interval; yrs., years; GCS, Glasgow Coma Scale; ACS, acute coronary syndrome. (PDF 39 kb) [file 12871_2019_683_MOESM5_ESM.pdf]

Table. Multivariate analysis of Fentanyl use

	OR_{adj} (95% CI)	
**Factor**	**Total**	**Trauma**
Surgeon	0.59 (0.46-0.77)	0.63 (0.46-0.87)
Age > 65 yrs	1.04 (0.87-1.25)	1.68 (1.31-2.15)
Female Patient	-	1.21 (0.95-1.54)
Patient intubated	39.16 (23.41-65.53)	52.39 (13.33-205.94)
GCS < 13	0.18 (0.11-0.28)	0.06 (0.02-0.17)
Trauma	11.97 (9.87-14.53)	n/a
ACS	0.13 (0.06-0.31)	n/a
Physician qualification resident	-	-
Physician sex female	1.59 (1.30-1.95)	1.66 (1.27-2.18)

Factors included in the multivariate analysis if $p < 0.05$ in univariate analysis; n/a, not applicable; -, not significant in univariate analysis; OR_{adj}, adjusted Odds-Ratio in multivariate analysis; CI, Confidence Interval; yrs, years; GCS, Glasgow Coma Scale; ACS, acute coronary syndrome.
