# Supplementary material for: Differences in pain treatment between surgeons and anaesthesiologists in a physician staffed prehospital emergency medical service: a retrospective cohort analysis
Source: BMC Anesthesiol. 2019 Jan 31;19:18. doi: 10.1186/s12871-019-0683-0 (PMC6357417; doi:10.1186/s12871-019-0683-0)
Supplement: Supplementary file 6 — Table Multivariate analysis of Morphine use. Factors included in the multivariate analysis if p < 0.05 in univariate analysis; n/a, not applicable; −, not significant in univariate analysis, ORadj adjusted Odds-Ratio in multivariate analysis, CI Confidence Interval, yrs. years, GCS Glasgow Coma Scale, ACS acute coronary syndrome. (PDF 38 kb) [file 12871_2019_683_MOESM6_ESM.pdf]

Table. Multivariate analysis of Morphine use

	OR_{adj} (95% CI)	
**Factor**	**Total**	**ACS**
Surgeon	0.78 (0.59-1.03)	0.88 (0.61-1.27)
Age > 65 yrs	1.21 (1.00-1.48)	-
Female Patient	0.86 (0.71-1.04)	-
Patient intubated	-	-
GCS < 13	0.54 (0.37-0.79)	-
Trauma	0.52 (0.34-0.78)	n/a
ACS	17.57 (14.28-21.61)	n/a
Physician qualification resident	1.62 (1.32-1.99)	1.82 (1.36-2.43)
Physician sex female	1.12 (0.90-1.39)	1.36 (1.01-1.84)

Factors included in the multivariate analysis if $p < 0.05$ in univariate analysis; n/a, not applicable; -, not significant in univariate analysis; GCS, Glasgow Coma Scale; ACS, acute coronary syndrome.
