# Supplementary material for: Differences in pain treatment between surgeons and anaesthesiologists in a physician staffed prehospital emergency medical service: a retrospective cohort analysis
Source: BMC Anesthesiol. 2019 Jan 31;19:18. doi: 10.1186/s12871-019-0683-0 (PMC6357417; doi:10.1186/s12871-019-0683-0)
Supplement: Supplementary file 3 — Standardized Bavarian EMS form for physicians DIVI version 5.1. (PDF 940 kb) [file 12871_2019_683_MOESM3_ESM.pdf]

Dieses Protokoll ist urheberrechtlich geschützt. Unveränderte Vervielfältigungen sind uneingeschränkt zulässig, jegliche kommerzielle Verwertung sowie jede Änderung bedarf der Genehmigung durch die DIVI (Deutsche Interdisziplinäre Vereinigung für Intensiv- und Notfallmedizin - www.divi.de)

Anaphylaktische Reaktion (jeweils schwerstes Symptom zählt): 0° lokal begrenzte Hautreaktion, I° Juckreiz, Flush, Urtikaria, Angioödem, II° wie I° + Nausea, Krämpfe, Erbrechen, Rhinorrhoe, Heiserkeit, Dyspnoe, Tachykardie, Hypotension III° wie II° + Defäkation, Larynxödem, Bronchospasmus, Zyanose, Schock IV° Atem-, Kreislaufstillstand

GCS für Kinder: Augen öffnen [4] spontan [3] auf Schreien [2] auf Schmerzreiz [1] keine Reaktion - Beste verbale Kommunikation [5] Plappern, Brabbeln [4] Schreien tröstbar [3] Schreien untröstbar [2] Stöhnen, unverständliche Laute [1] keine Reaktion - Beste motorische Reaktion [6] Spontane Bewegungen [5] auf Schmerzreiz gezielt [4] auf Schmerzreiz normale Beugeabwehr [3] auf Schmerzreiz abnorme Abwehr [2] Strecksynergismen [1] keine Reaktion

Pat-Stammdaten (ggf. mit Aufkleber überkleben / überdrucken)

Name*
Vorname*
Geb.Dat.* ☐ *Pseudonym
Straße
Plz, Ort
Kasse / Nr.
Vers. Nr.

Geschlecht ○ männlich ○ weiblich — BMI (Gewicht [kg] / (Größe [m])²) ○ ≤ 40 ○ > 40 — Alter ___ Jahre ○ 1-7 Tg ○ 8-28 Tg

NOTARZT-EINSATZPROTOKOLL Version 5.1

Empfehlung der DIVI 2015 (www.divi.de)

○ Einsatz Nr. ○ Pat./Auftr. Nr. — Pers Nr.
Standort — Leitstelle (KFZ-Kennz.)

Einsatztechnische Daten

Beteiligt: ☐ NEF ○ Selbstfahrer ☐ NAW ☐ RTW ☐ KTW ☐ RTH ☐ ITH ☐ sonst. ☐ Bergrettung ☐ Wasserrettung

Einsatz-Datum
Einsatz-Ort: Straße / Plz / Ort
○ Wohnung ○ Altenheim ○ Arbeitsplatz ○ Sportstätte ○ Arztpraxis ○ Krankenhaus ○ Geburtshaus ○ öff. Raum ○ Straße ○ Schule ○ Bildungseinrichtung ○ Massenveranstaltung ○ Sonstige

Einsatz-Art: ○ Primäreinsatz ○ Folgeeinsatz ○ Fehleinsatz ○ Notarzt-Nachforderung ○ Sekundäreinsatz z.B. Verlegung ○ vorsorgliche Bereitstellung
○ kein Patient ○ abbestellt ○ Pat. bereits abtransportiert ○ böswillige Alarmierung
Einsatzabbruch ○ technische Gründe ○ Wetter ○ Sonstige

Transportziel
☐ Voranmeldung ○ Stroke Unit ○ Herzkatheter ○ Traumazentrum

Symptom-Beginn (z.B. Kollaps Unfallzeitpunkt) ☐ vor über 24h ☐ Kollaps beobachtet
Alarm
Ankunft (Einsatzort)
Ankunft (am Pat.) ☐ vor Rettungsdienst
Abfahrt
Übergabe
Einsatzbereit
Ende

○ Arzt in Weiterb. ○ Facharzt ⇨ ☐ Anästhesie ☐ Chirurgie ☐ Innere ☐ Andere ☐ Pädiatrie ☐ Neurologie ☐ Allg. Medizin ☐ Zusatz Intensiv

Notarzt
Assistenz — ○ Rett.Ass. ○ Rett.San. ○ Notfall San. ○ (Int.)Pfleg.
RTW-Team — Rufname eigenes Fahrzeug

Notfallgeschehen, Anamnese, Erstbefund, Vormedikation, Vorbehandlung

AZ des Pat. vor dem Ereignis ○ gesund ○ krank uneingeschränkt ○ leicht eingeschränkt ○ schwer eingeschränkt ○ akut lebensgefährdet ○ unbekannt
Ersthelfermaßnahmen (Laien) ○ suffizient ○ insuffizient ○ keine
First Responder ☐ vor Ort - ggf. Uhrzeit des Eintreffens
☐ Besiedelung mit multiresistenten Keimen (MRSA, MRSE, ESBL etc) vorbekannt ☐ abgedeckt offen

Erstbefunde - Neurologie

○ ohne path. Befund — Zeitpunkt

Kinder GCS ⇦

Augen öffnen
- spontan (4)
- auf Aufforderung (3)
- auf Schmerzreiz (2)
- kein Augenöffnen (1)

beste verbale Reaktion
- konversationsfähig
  - orientiert (5)
  - desorientiert (4)
- inadäquate Äußerungen (3)
- unverständliche Laute (2)
- keine (1)

beste motorische Reaktion (re / li; Arm / Bein)
- folgt Aufforderung (6)
- gezielte Abwehr (5)
- ungezielte Abwehr (4)
- Beugesynergismen (3)
- Strecksynergismen (2)
- keine (1)

Glasgow Coma Scale (Summe)
Zur Summierung der GCS wird nur der Wert der Extremität mit der besten motorischen Reaktion verwendet

Extremitätenbewegung (re / li; Arm / Bein)
- normal (1)
- leicht vermindert (2)
- stark vermindert (3)

Bewusstseinslage
○ nicht beurteilbar ○ wach ○ getrübt ○ bewusstlos ☐ Reakt. auf Ansprache ☐ Reakt. auf Schmerzreiz ☐ analgosediert / Narkose

Neurologische Auffälligkeiten
○ ohne path. Befund ○ nicht beurteilbar
☐ Seitenzeichen (Pupillen, periph. Motorik)
☐ kein Lächeln ☐ Sprachstörung
☐ Sehstörung ☐ Demenz
☐ Querschnittssymptomatik
☐ Babinski Zeichen ☐ Meningismus
☐ vorbestehende neurologische Defizite
☐ Sonstige

Pupillenweite — rechts: ○ eng ○ mittel ○ weit ○ entrundet — links: ○ eng ○ mittel ○ weit ○ entrundet

Lichtreaktion — rechts: ○ prompt ○ träge ○ keine — links: ○ prompt ○ träge ○ keine

Messwerte initial

○ keine — Schmerzen 0 1 2 3 4 5 6 7 8 9 10
RR ___/___ HF ___ BZ ___ LOW HIGH
○ rhythmisch ○ arrhythmisch ○ mg/dl ○ mmol/l
AF SpO₂ Temp etCO₂
○ bei Raumluft ○ unter O_2-Gabe

EKG ○ kein EKG
○ Sinusrhythmus ○ Abs. Arrhythmie ○ AV-Block II° ○ AV-Block III° ○ Schrittmacherrhythmus ○ Kammerflimmern ○ PEA / EMD ○ Asystolie ☐ STEMI ☐ schmale QRS-Tachykardie ☐ breite QRS-Tachykardie ☐ SVES / VES monomorph ☐ VES polymorph

Atmung ○ nicht US
○ unauffällig ○ Dyspnoe ○ Schnappatmung ○ Apnoe ○ Beatmung ☐ Stridor ☐ Zyanose ☐ Spastik ☐ Hyperventilation ☐ Atemwegsverlegung ☐ Rasselgeräusche
○ sonstiges path. Atemmuster (Biot, Cheyne Stokes etc.)

Haut ○ nicht US
○ unauffällig ○ pathologisch ☐ stehende Hautfalten ☐ Dekubitus ☐ Exanthem ☐ Oedeme ☐ kaltschweißig

Psyche ○ nicht US
☐ unauffällig ☐ nicht beurteilbar ☐ wahnhaft ☐ erregt ☐ depressiv ☐ verwirrt ☐ aggressiv ☐ ängstlich ☐ suizidal ☐ verlangsamt/stuporös ☐ euphorisch ☐ motorisch unruhig

Erkrankungen

○ keine

ZNS
☐ Schlaganfall, TIA, intrakranielle Blutung
☐ im Lysefenster (Symptombeginn erfassen!)
☐ Krampfanfall ☐ Status epilepticus
☐ Meningitis / Encephalitis
☐ Synkope
☐

Herz-Kreislauf
☐ Akutes Koronarsyndrom
☐ STEMI ☐ Vorderwand ☐ Hinterwand
☐ Rhythmusstörung ☐ tachy ☐ brady
☐ Lungenembolie
☐ orthostatische Fehlregulation
☐ Herzinsuffizienz ☐ Lungenödem
☐ hypertensiver Notfall / hypertensive Krise
☐ kardiogener Schock
☐ Schrittmacher- / ICD-Fehlfunktion
☐

Atmung
☐ Asthma (Anfall) ☐ Status asthmaticus
☐ COPD (ggf. Exazerbation)
☐ Heimbeatmung ☐ Heimsauerstoff
☐ Pneumonie, Bronchitis
☐ Hyperventilationssyndrom
☐ Aspiration
☐ Haemoptysen
☐

Abdomen
☐ Akutes Abdomen
☐ GI-Blutung ☐ obere ☐ untere
☐ Kolik (z.B. Niere, Galle)
☐ Enteritis
☐

Psychiatrie
☐ Psychose, Manie, Erregungszustand
☐ Angst, Depression
☐ Intoxikation ⇨ ☐ akzidentell ☐ Alkohol ☐ Drogen ☐ Medikamente ☐ Sonstiges
☐ Entzug, Delir
☐ Suizid(versuch)
☐ Psychosoziale Krise
☐

Stoffwechsel
☐ Hypoglykämie
☐ Hyperglykämie
☐ Exsiccose
☐ Urämie/ANV ☐ bek. Dialysepflichtig
☐

Pädiatrie
☐ Fieberkrampf
☐ Pseudokrupp
☐ SIDS / Near-SIDS
☐

Gynäkologie
☐ Schwangerschaft
○ drohende Geburt ○ präklinische Geburt
☐ (Prä-)Eklampsie
☐ vaginale Blutung
☐

Sonstige
☐ anaphylakt. Reaktion ○ I-II° ○ III-IV°
☐ Hitzeerschöpfung, Hitzschlag
☐ Unterkühlung / Erfrierung
☐ hochfieb. Infekt / Sepsis / sept. Schock
☐ Influenza ☐ Hepatitis / HIV
☐ akute Lumbago ☐ Epistaxis
☐ soziales Problem (ohne psych. Störung)
☐ medizinische Behandlungskomplikation
☐

Verletzungen

○ keine — Zusammenhang mit sportlicher Aktivität ○ Ja ○ Nein; beruflicher Aktivität ○ Ja ○ Nein

	leicht	mittel	schwer	geschlossen	offen
Schädel-Hirn	☐	☐	☐	☐	☐
Gesicht	☐	☐	☐	☐	☐
Hals	☐	☐	☐	☐	☐
Thorax	☐	☐	☐	☐	☐
Abdomen	☐	☐	☐	☐	☐
Wirbelsäule	☐	☐	☐	☐	☐
Becken	☐	☐	☐	☐	☐
Obere Extremitäten	☐	☐	☐	☐	☐
Untere Extremitäten	☐	☐	☐	☐	☐
Weichteile	☐	☐	☐	☐	☐

☐ Polytrauma.

☐ Verbrennung, Verbrühung ___ Grades ___ % ___ Grades ___ %
☐ Verätzung
☐ Verschüttung ☐ Einklemmung
☐ Inhalationstrauma ☐ Elektrounfall
☐ BeinaheErtrinken ☐ Tauchunfall
☐ haemorrhagischer Schock
☐ Sonstige

Unfallmechanismus
Trauma ○ stumpf ○ penetrierend
Sturz ○ <3m ○ >3m
Verkehr: Pat war ○ Fußgänger ○ Motorradfahrer ○ PKW-/LKW-Insasse ○ Fahrradfahrer ○ Sonstige
Gewaltanwendung ☐ Schlag ☐ Stich ☐ Gewaltverbrechen ☐ Schuss ☐ Sonstige

Erstdiagnosen

NACA SCORE ○ I (geringfügige Störung) ○ II (leichte Störung) ○ III (mäßige Störung) ○ IV (Lebensgefahr nicht auszuschließen) ○ V (akute Lebensgefahr) ○ VI Reanimation ○ VII (Tod) ☐ Palliative Situation

Verlaufsbeschreibung

Zeit / Maßnahmen
Puls, RR, HDM, Defi, Transport T-T, Intubation, Injektion bei Rea: Suprarenin S, Amiodaron A

Skala: ≥220, 200, 180, 160, 140, 120, 100, 80, 60, ≤40

Werte über 220 bzw. unter 40 werden in der obersten bzw. untersten Zeile zusammen mit dem zugehörigen Zeichen für Blutdruck oder Puls (∨ bzw. ○) als Zahl eingetragen

O2 [l/min]
SpO2 [%]
etCO2 [mmHg]

Medikation

○ keine Medikation

Wirkstoff / Handelsname / Infusion	Dosis / Dosen	Wirkstoff / Handelsname / Infusion	Dosis / Dosen

...Vorab-Gabe durch Rettungsdienst (Notkompetenz): ☐ Adrenalin ☐ Amiodaron ☐ Glucose ☐ ß-Mimetikum ☐ Analgetikum ☐ Benzodiazepin ☐ Nitrospray ☐ Sonstige

Lysetherapie ○ vor Kreislaufstillstand ○ während Kreislaufstillstand ○ nach ROSC — Beginn...

Reanimation / Tod / Todesfeststellung (ergänzende Angaben)

☐ Reanimation ○ ROSC im Verlauf ○ niemals ROSC ○ erfolglos
Beginn Rea: ○ Ersthelfer ○ First Resp. ○ Rettungsd. ○ Notarzt
☐ keine Reanimation ○ nicht gewünscht / Patientenverfügung ○ zu spät ○ aussichtslose Grunderkrankung ○ Sonstige
☐ KH-Aufnahme ○ mit ROSC ○ laufende Reanimation

☐ **Tod** ☐ Todesfeststellung ○ natürlich ○ unklar ○ nicht natürlich
Todeszeitpunkt ☐ Todeszeitpunkt nicht bestimmbar

Übergabe

○ keine — Zeitpunkt — Glasgow Coma Scale
RR ___/___ HF ___ BZ ___ LOW HIGH
○ rhythmisch ○ arrhythmisch ○ mg/dl ○ mmol/l
AF SpO₂ Temp etCO₂
Schmerzen 0 1 2 3 4 5 6 7 8 9 10

EKG ○ kein EKG
○ Sinusrhythmus ○ Abs. Arrhythmie ○ AV-Block II° ○ AV-Block III° ○ Schrittmacherrhythmus ○ Kammerflimmern ○ PEA / EMD ○ Asystolie ☐ STEMI ☐ schmale QRS-Tachykardie ☐ breite QRS-Tachykardie ☐ SVES / VES monomorph ☐ VES polymorph

Atmung ○ nicht US
○ unauffällig ○ Dyspnoe ○ Schnappatmung ○ Apnoe ○ Beatmung ☐ Stridor ☐ Zyanose ☐ Spastik ☐ Hyperventilation ☐ Atemwegsverlegung ☐ Rasselgeräusche
○ sonstige pathologische Atemmuster (Biot, Cheyne Stokes etc.)

Bewusstsein ○ nicht US
○ wach ○ getrübt ○ bewusstlos ○ nicht beurtb. ☐ Reakt. auf Ansprache ☐ Reakt. auf Schmerzreiz ☐ analgosediert / Narkose

Massnahmen

Zugänge — Art / Ort / Größe — Anzahl
☐ peripherer Zugang ☐ bereits vorhanden
☐ intraossäre Punktion
☐ Transnasal-Applikator
☐ Sonstige
☐ Zugang erschwert ○ > 2 Versuche ○ Zugang unmöglich → Verfahrenswechsel

Atemweg
☐ Sauerstoffgabe ___ l/min ○ als Präoxygenierung
☐ Freimachen der Atemwege / Absaugen
☐ Masken-/Beutel-Beatmung ☐ kontrolliert ☐ unterstützend, Demand-Ventil ☐ nicht möglich
☐ supraglottische Atemwegshilfe ○ Larynxmaske ○ Larynxtubus ○ Sonstige
☐ endotracheale Intubation ○ oral ○ nasal — Größe [mm]
☐ fiberoptische / elektronische Intubationshilfe
☐ Koniotomie / chirurgischer Atemweg
☐ sonstiger Atemwegszugang
☐ Intubation erschwert — Anzahl Versuche

Beatmung
○ manuell ○ maschinell ○ kontrolliert ○ assistiert ○ NIV — FiO2 — AF — AMV — PEEP — P_{max}

Defibrillation
☐ AED ☐ Defi — Erstanwendung ○ Laien ○ First Resp. ○ Rettungsdienst ○ Arzt — ○ monophasisch ○ biphasisch
1. Defibrillation (Zeitpunkt) — 1. ROSC (Zeitpunkt)
Anzahl Defi insgesamt — $Energie_{max}$ Joule

Reanimation (ergänzende Angaben im Kasten „Reanimation / Tod / Todesfeststellung“)
☐ Herzdruckmassage — Beginn HDM:
○ Feedbacksystem ○ mechanisches Thoraxkompressionssystem
☐ Aktive Kühlung ○ Infusion ○ Kühlpackungen ○ technisch
☐ Vorab: Telefonanleitung zur Reanimation

Sonstige
☐ Spezielle Lagerung ☐ Zervikalstütze ☐ Vakuumschiene ☐ Vakuummatratze ☐ Schaufeltrage ☐ Spineboard ☐ stabile Seitenlage ☐ Schocklagerung ☐ Oberkörper Hochlagerung ☐ Flachlagerung ☐ Sitzend ☐ Sonstige…….. ☐ Wärmeerhalt ☐ Inkubator
☐ 12-Kanal EKG ☐ Spritzenpumpe(n) ☐ Funkübermittlung ☐ invasiver RR ☐ externer Schrittmacher ☐ Kardioversion
☐ Verband ☐ Reposition ☐ Beckenschlinge
☐ Thoraxdrainage re. ☐ Thoraxdrainage li. ☐ Blasenkatheter
☐ Magensonde ☐ Ultraschall
☐ Entbindung ☐ Krisenintervention
☐ Sonstiges

Einsatzverlauf - Besonderheiten

○ Übernahme aus arztbesetztem Rettungsmittel
○ Übergabe an arztbesetztes Rettungsmittel
○ Transport ins Krankenhaus ☐ mit Arzt ☐ RTH
☐ mindestens eine Klinik nimmt nicht auf
☐ Zwangsbelegung ☐ Zwangsunterbringung
○ nur Untersuchung und Behandlung
○ Patient lehnt Transport ab
○ Patient nicht transportfähig
○ Therapieverzicht / -beschränkung bewusst
☐ Erhöhter Hygieneaufwand
☐ erschwerter Pat-Zugang ☐ techn. Rettung
☐ Schwerlasttransport erforderlich
☐ LNA am Einsatz
☐ MANV ☐ Behandlung mehrerer Patienten

Übergabe

○ keine ○ Einsatzstelle ○ ZNA / INA ○ Stroke Unit ○ Intensivstation ○ OP direkt ○ Praxis ○ Sonstige
○ Hausarzt / KV-Arzt vor Ort ○ Fachambulanz ○ Schockraum ○ Herzkatheterlabor ○ CPU ○ Allgemeinstation

Übergabe an (Name)

Bemerkungen (z.B. Verlauf, Hausarzt, Telefon-Nummer Angehörige, Notkompetenz-Massnahmen)

Unterschrift Notarzt
